# Assessment of burnout, resilience, and thriving among academic health professionals: findings from an international study

**DOI:** 10.3389/fpubh.2024.1366612

**Published:** 2024-04-05

**Authors:** Abdul Rahman Fata Nahas, Mohamed Hassan Elnaem, Naeem Mubarak, Merna Abou Khatwa, Muna Barakat, Erwin Faller, Lamyaa M. Kassem, Diana Laila Ramatillah, Ammar Jaber, Muhammad Eid Akkawi, Abdulkareem Mohammed Al-Shami, Sarath Chandran, Islam Mohamed, Iain Jack, Ahmed Abouelhana, Aaron Courtenay, Mahmoud E. Elrggal

**Affiliations:** ^1^Department of Pharmacy Practice, Faculty of Pharmacy, International Islamic University Malaysia, Kuantan, Pahang, Malaysia; ^2^School of Pharmacy and Pharmaceutical Sciences, Ulster University, Coleraine, United Kingdom; ^3^Department of Pharmacy Practice, Lahore Medical and Dental College, University of Health Sciences, Lahore, Pakistan; ^4^Department of Clinical Pharmacy and Pharmacy Practice, Faculty of Pharmacy, Alexandria University, Alexandria, Egypt; ^5^Department of Clinical Pharmacy and Therapeutics, Faculty of Pharmacy, Applied Science Private University, Amman, Jordan; ^6^School of Allied Health Sciences, Pharmacy Department, San Pedro College, Davao City, Philippines; ^7^Department of Pharmacy Practice, Unaizah College of Pharmacy, Qassim University, Unaizah, Qassim, Saudi Arabia; ^8^Faculty of Pharmacy, Universitas 17 Agustus, Jakarta, Indonesia; ^9^Department of Clinical Pharmacy and Pharmacotherapeutics, Dubai Pharmacy College, Dubai, United Arab Emirates; ^10^University College MAIWP International (UCMI), Kuala Lumpur, Malaysia; ^11^College of Pharmaceutical Sciences, Government Medical College, Kannur, India; ^12^California Northstate University College of Medicine, Elk Grove, CA, United States; ^13^Faculty of Medicine, Al-Qunfudah, Umm Al-Qura University, Saudi Arabia

**Keywords:** burnout, resilience, thriving, academic, health professions

## Abstract

**Introduction:**

Burnout, resilience, and thriving significantly impact academics, particularly in health professions, where responsibilities are extensive. This study aimed to explore these constructs among academic health professionals, examining sociodemographic and work-related factors influencing these outcomes.

**Methods:**

A cross-sectional study was conducted among academic health professionals via web-based professional networks from August 2022 to February 2023. Validated tools were used, and descriptive and inferential statistics were applied.

**Results:**

505 participants were included, predominantly female (63%), with a mean age of 38.15 ± 9.6 years. High burnout was reported by 10.9%, 13.7% experienced exhaustion, and 6.3% were disengaged. Resilience and thriving were moderate at 59.2 and 51.9%, respectively. Age correlated negatively with burnout (*r* = −0.131, *p* = 0.003) but positively with resilience (*r* = 0.178, *p* < 0.001). Females reported higher exhaustion (*p* = 0.014), while males showed greater resilience (*p* = 0.016). Instructors exhibited lower resilience compared to assistant professors (*p* < 0.001) and associate professors (*p* < 0.001). Those at public universities reported higher exhaustion than those at private universities (*p* < 0.001).

**Conclusion:**

Variable levels of burnout, resilience, and thriving were observed among academic health professionals, influenced by sociodemographic and work-related factors. Interventions targeting resilience and thriving may mitigate burnout risk and enhance engagement among academics in health professions.

## 1 Introduction

In the era of globalization and the advancement of technologies, the work environment has become more competitive and demanding, with work overload and stress imposed on workers, and academics are no exception. The disparities between the enduring and demanding work requirements and the capacity of academicians to tackle these demands ultimately culminate in burnout and emotional distress (1). Professional burnout is a psychological state that negatively impacts one’s relationship with one’s work, resulting in emotional exhaustion, reduced job engagement, commitment, and inappropriate practice within the work environment (2). Worldwide, the prevalence of burnout and emotional distress among academicians is relatively high, affecting over one-third of the population (3, 4). Furthermore, a high proportion of burnout tends to be affected by work type, chronic disease, and gender, reflecting other dimensions of this issue in academia (5).

Health field academicians face burnout because of a lack of coping with and adapting to ongoing stressful challenges that are considered part of academic job portfolios. Coping can be apparent via a worker’s ability to be resilient and thrive following stressful events. Resilience is the ability to rebound after facing adversity via effective adaptation or management of substantial stressful events (6). By contrast, thriving refers to a positive mindset in dealing with work-related stressors with a joint state of vitality and learning, resulting in a higher level of functioning (7). There is a clear distinction between resilience and thriving in the workplace, particularly in the aftermath. Resilience results in a return to a balanced state, while thriving leads to significant gains. Those who exhibit determination are more likely to foster thriving, resulting in improved stress resilience (6).

Several possible risk factors may predispose health field academics to burnout and stress, such as increased workload, a persistent requirement to secure research grants, reduced resources for self-development, and increased demands on building capacities to cope with the ongoing trend to maintain e-learning activities (3, 5). Burnout is attributed mainly to the work environment, demands, and struggle to achieve work-life balance (8). Previous research examined the relationship between coping styles and burnout among healthcare professionals and found that task-oriented and adaptive coping was associated with a decreased risk of burnout, potentially improving staff well-being (9, 10). Moreover, individual-level coping capacities and handling workplace dynamics have also been found to shape the risk of stress and burnout and affect mental well-being among academic individuals (1, 11). Several recent reports have highlighted the ongoing risk of burnout in academia, resulting in a quiet quitting phenomenon in some cases that might affect the long-term sustainability of high-quality education (12).

Although there is some country-specific data on burnout assessment among academicians (3, 13, 14), this issue was not commonly assessed together with other relevant traits, such as resilience and thriving, from international perspectives. Therefore, assessing academic burnout and coping abilities in the health field is crucial to determining the most effective ways to reinforce academic success and mental well-being. Considering this, this study aimed to investigate the phenomenon of academic burnout, resilience, and thriving among academic health professionals while examining various sociodemographic and work-related factors that may impact these outcomes.

## 2 Methods

This was a cross-sectional, survey-based study conducted among international academic health professionals. Data were collected from October 2022 to February 2023 based on convenience sampling. A self-administered online pre-validated survey was prepared in Google Forms and distributed via participants’ email addresses and social and professional networks. Regular reminders were sent every 2 weeks to ensure adequate response rate.

### 2.1 Ethical approval

The Research and Ethics Committee at the International Islamic University Malaysia (IIUM) approved the study protocol (IREC 2022-391). All study participants provided their online consent before their participation. The consent form was placed on the introductory page of the study form with all relevant details about the study and voluntary participation. Participants were asked to read these details carefully and give their consent if they wanted to continue participating in the research and start answering the survey questions.

### 2.2 Sample size and sampling

In order to calculate the required sample size, the study follows the method suggested by Krejcie and Morgan (1970) where a minimum of 376 participants is needed to ensure acceptable statistical power. The inclusion criteria included all consented academic health professionals with verified professional identities on social networks like LinkedIn. Participants were eligible to participate if they were employed on academic appointments in one of the accredited academic programs in health professions in any country. Participation was voluntary and not associated with any compensation or incentives.

### 2.3 Measures

The survey consisted of four main parts:

Part 1: Sociodemographic, such as country, age, gender, marital status, and having kids.
Part 2: Work-related factors, such as annual salary, employment status, academic rank, institution type, experience, and loads of teaching and research.
Part 3: Oldenburg Burnout Inventory (OLBI):
Oldenburg Burnout Inventory has been regarded as a reliable and robust tool to measure academic burnout (15–17). It effectively measures burnout by assessing all aspects of exhaustion, namely physical, affective, and cognitive. This suits those whose jobs involve thinking and mental functioning (18). OLBI consists of two subscales: Exhaustion (OLBI-E) and Disengagement (OLBI-D), each with eight questions. To interpret the OLBI scores for our sample, we divided the frequency distributions of the mean scores for OLBI-E and OLBI-D into quartiles. Accordingly, OLBI-E scores of more than 22 were considered high exhaustion, whereas, for disengagement, high disengagement was deemed if OLBI-D scores were more than 21. The scores were then categorized into “low,” “average,” and “high” scores. This yielded four groups, i.e., High burnout (participants with “high” scores for both OLBI-E and OLBI-D), Exhaustion and Disengagement, either Disengaged or Exhausted (participants who had a “high” score for that particular subscale combined with a “low” or “average” score on the other subscale), and a fourth group of low-burnout (participants with “low” or “average” scores for both subscales) (19). Table 1 details OLBI subscales score interpretation and grouping.
Part 4: Stress Adaptation Scale (SAS).
Stress Adaptation Scale is a valid and reliable scale to assess two critical individual capacities: resilience and thriving. It consists of two subscales: the Brief Resilience Scale (BRS) and the Brief Thriving Scale (BTS). Each subscale consisted of six statements. Answers are based on a five-point Likert scale ranging from strongly disagree (1) to strongly agree (5). Scores are interpreted by summing the total score of the subscale and dividing it by six (i.e., the number of statements) to get the mean value. Scores are then categorized into five groups based on mean values (6, 20). Table 2 details the interpretation and grouping of the BRS and BTS scores.

**Table 1:** OLBI subscales score severity and burnout grouping.

	OLBI-exhaustion scores	OLBI-disengagement scores
High (top quartile)	>22	>21
Average	18–22	16–21
Low (bottom quartile)	<18	<16
High burnout group	High	High
Exhausted group	High	Low or Average
Disengaged group	Low or Average	High
Low-burnout group	Low or Average	Low or Average

**Table 2:** Grouping based on BRS and BTS score ranges.

	Score ranges (mean)	
	BRS	BTS
Very high	5.00–4.67	5.00–4.83
High	4.50–4.00	4.67–4.33
Medium	3.83–3.00	4.17–3.33
Low	2.83–2.17	3.17–2.50
Very low	2.00–1.00	2.33–1.00

### 2.4 Reliability of the used scales

Cronbach’s alpha (α) measures the internal consistency of an assessment instrument. A Cronbach’s alpha value between 0.6 and 0.8 is considered acceptable (21). A pilot study was conducted among 35 participants representing our population to ensure the reliability of OLBI and SAS among our sample. The Cronbach’s alpha values of OLBI and SAS subscales were greater than 0.6, with OLBI-E at 0.82, and OLBI-D at 0.64, BRS at 0.61, and BTS at 0.9. Therefore, both scales demonstrated adequate overall internal consistency.

### 2.5 Data analysis

The responses were downloaded into an Excel® spreadsheet. The data were then imported for analysis to the Statistical Package for Social Sciences (SPSS, IBM, United States), version 25.0. Descriptive and inferential analyses were performed whenever appropriate. A P value of ≤ .05 was considered significant.

## 3 Results

### 3.1 Sociodemographic and work-related characteristics of the study participants

A total of 505 participants were included in the study. Participants ranged in age from 22 to 80, with a mean of 38.15 ± 9.6 years. Most of our sample consisted of female participants (63%) who were married (65.1%) and had kids (62.4%). Most respondents were from Egypt (19.4%) and India (17%), and a considerable number were also collected from Pakistan (12.1%), Jordan (11.1%), and the Philippines (10.7%).

Regarding the work-related characteristics of the study participants, the majority had an annual salary of less than U$25,000 (66.3%) and were permanent lecturers (69.5%). 14.7% were professors, and only 5% were from public colleges; however, the majority were from pharmacy faculties (67.1%), and 42.6% had more than 10 years of working experience. Over one-third of the sample (38.8%) had no clinical-related work, 13.7% were not involved in research, and 17.4% were involved in a 6-h weekly postgraduate supervision. Although about half of the participants had no administration post (56.6%), only 26.9% stated they were not involved in administration work. Nearly half of the study sample (51.1%) were familiar with the remote learning software available in their institution. However, less than half of the sample (41.2%) considered the available remote learning resources sufficient. Table 3 shows the sociodemographic and work-related characteristics of our sample.

**Table 3:** Participants’ sociodemographic and work-related characteristics (*N* = 505).

	*N*	%
Gender		
Male	187	37.0
Female	318	63.0
Marital status		
Divorced/Separated	22	4.4
Married	329	65.1
Single	145	28.7
Widowed	9	1.8
Having kids		
No	190	37.6
Yes	315	62.4
Country		
Egypt	98	19.4
India	86	17.0
Pakistan	61	12.1
Jordan	56	11.1
Philippine	54	10.7
KSA	40	7.9
Indonesia	34	6.7
UAE	31	6.1
Malaysia	19	3.8
Syria	10	2.0
Others (Yemen, Sudan, Libya, Ethiopia, Iraq, Tunisia, and Afghanistan)	16	3.2
Annual salary		
< U$25 K	335	66.3
U$25 K to < U$50 K	79	15.6
U$50 K to < U$100 K	67	13.3
> U$100 K	24	4.8
Employment status		
Visiting lecturer	30	5.9
Permanent lecturer	351	69.5
Contract lecturer	124	24.6
Academic rank		
Instructor/Teaching assistant	108	21.4
Lecturer	116	23.0
Assist. Prof.	101	20.0
Assoc. Prof.	106	21.0
Prof.	74	14.7
Institution type		
Private college	155	30.7
Public college	28	5.5
Private Unit	160	31.7
Public Unit	162	32.1
Field/Faculty		
Allied Health/Medical Lab	31	6.1
Public health	11	2.2
Nursing/Midwifery	26	5.1
Pharmacy	339	67.1
Dentistry	28	5.5
Medicine	70	13.9
Working experience in years		
<2 years	66	13.1
2–5 years	95	18.8
>5–10 years	129	25.5
>10 years	215	42.6
How many working hours per week do you spend in the clinical setting?		
None	196	38.8
< 5 h	87	17.2
>5–10 h	112	22.2
> 10 h	110	21.8
How many hours do you usually spend on research in a week?		
None	69	13.7
< 10 h	265	52.5
>10–20 h	124	24.6
> 20 h	47	9.3
How many hours of postgraduate supervision (e.g., student meetings and discussions) do you usually spend in a week?		
None	167	33.1
<3 h	102	20.2
>3–6 h	148	29.3
>6 h	88	17.4
Do you currently hold an administrative post? (e.g., Dean, Deputy Dean, Head of Department, and Coordinator)		
No	286	56.6
Yes	219	43.4
How many hours of administrative work do you usually spend in a week?		
None	136	26.9
<3 h	89	17.6
>3–6 h	124	24.6
>6 h	156	30.9
How familiar are you with remote learning software?		
No	38	7.5
Not much	109	21.6
Familiar	258	51.1
Very	100	19.8
Does your institution provide sufficient remote learning resources (software, workshops, and equipment)		
Insufficient	95	18.8
Partially sufficient	202	40.0
Sufficient	208	41.2

### 3.2 Burnout assessment based on OLBI score

**Figure 1:**
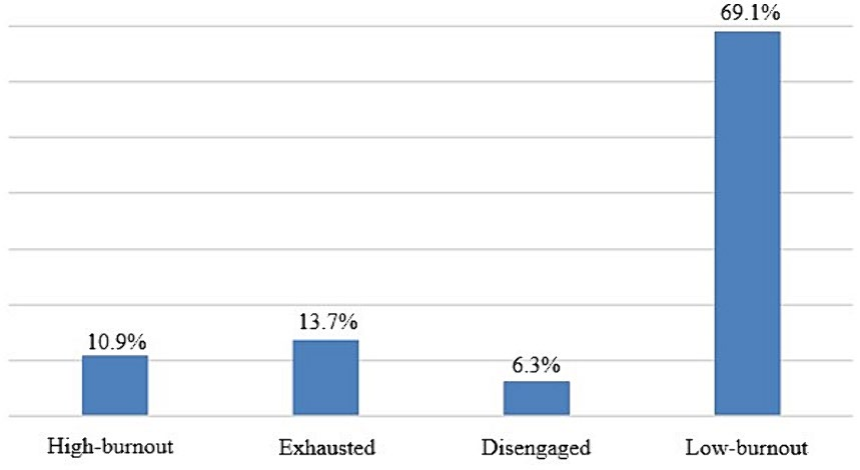
OLBI grouping of the study sample expressed in proportions.

Based on OLBI scoring and burnout grouping (Table 1), our analysis showed that 55 (10.9%) respondents had high burnout, 69 (13.7%) were exhausted, and 32 (6.3%) were disengaged (Figure 1). Our sample had an average exhaustion level (M = 20.0 ± 3.7) and an average disengagement level (M = 18.7 ± 3.4).

### 3.3 Resilience and thriving assessment based on BRS and BTS

**Figure 2:**
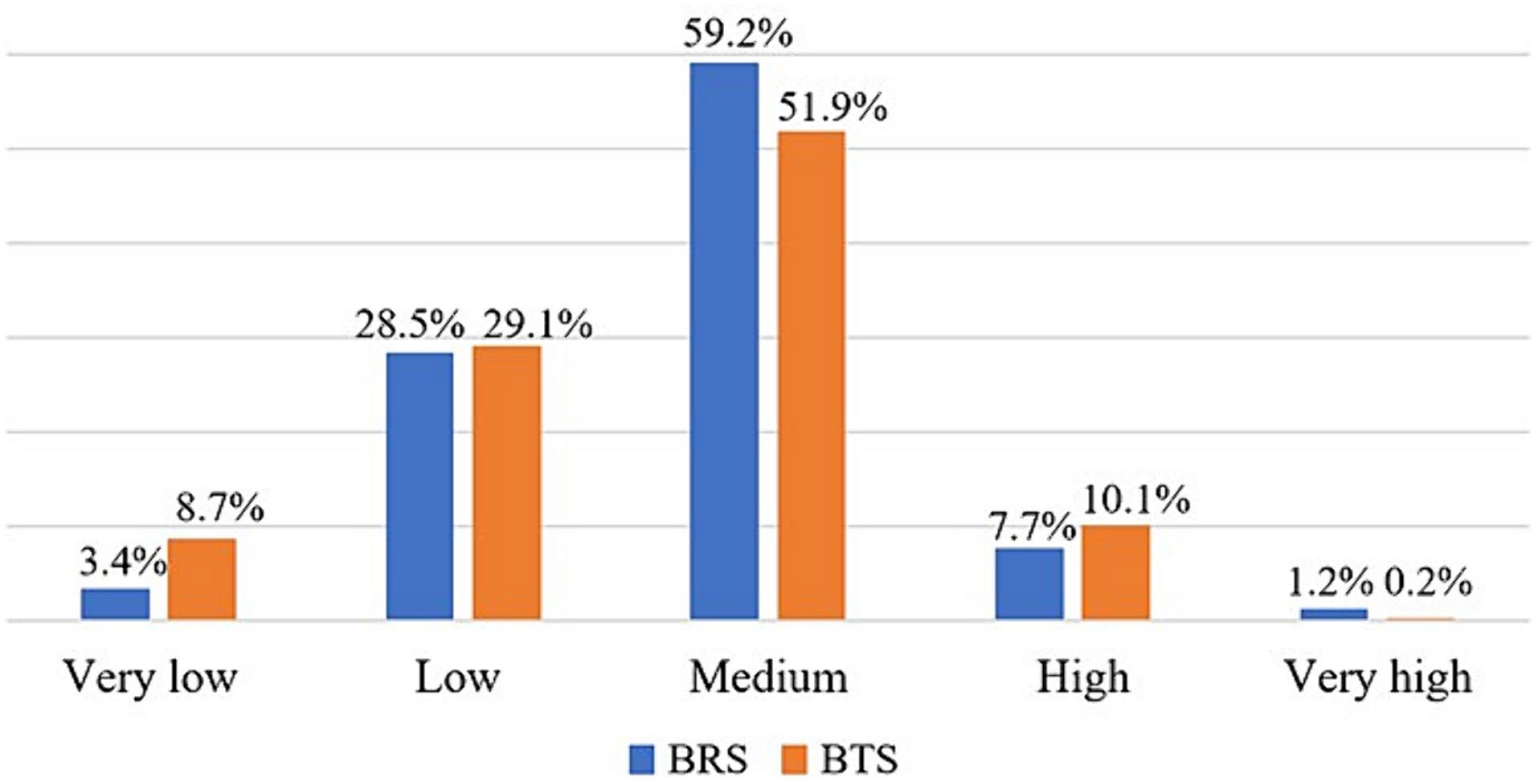
BRS and BTS grouping of the study sample expressed in proportions.

The proportion of participants with BRS scores in the medium range was 59.2% (*n* = 299), translating into an overall medium resilience of our sample with a mean BRS score of 3.14 ± 0.6. Similarly, the proportion of participants with BTS scores in the range considered medium was 51.9% (*n* = 262), which also translates into an overall medium thriving of our sample with a mean BTS score of 3.44 ± 0.7. Figure 2 shows more details on the BRS and BTS analysis.

### 3.4 Inferential analysis of demographic and work-related characteristics, burnout, resilience, and thriving

#### 3.4.1 Age and gender

Age was significantly and negatively correlated with OLBI-E scores (*r* = −0.131, *p* = 0.003) but positively with BRS scores (*r* = 0.178, *p* = 0.001). An independent-sample *t*-test showed that gender differs significantly in OLBI-E and BRS scores. Females were more exhausted (M = 20.35 ± 3.57) compared to males (M = 19.50 ± 3.96, *p* = 0.014), whereas males were more resilient (M = 3.22 ± 0.57) than females (M = 3.09 ± 0.58, *p* = 0.016).

#### 3.4.2 Employment status, academic rank, and responsibilities

There was a significant difference in employment status, in which contract lecturers (Md [IQR] = 19.50 [4.00]) were less exhausted than permanent lecturers (Md [IQR] = 20.00 [5.00], *p* = 0.028). A significant difference in academic ranking was also revealed. Instructors were more exhausted (Md [IQR] = 21.00 [5.00]) than lecturers (Md [IQR] = 20.00 [3.00], *p* < 0.001) and assistant professors (Md [IQR] = 20.00 [4.00], *p* = 0.043), but less exhausted than associate professors (Md [IQR] = 19.00 [6.00], *p* < 0.001) and professors (Md [IQR] = 20.00 [6.00], *p* = 0.044). Instructors were also less resilient (Md [IQR] = 3.00 [0.67]) than assistant professors (Md [IQR] = 3.00 [0.83], *p* < 0.001) and associate professors (Md [IQR] = 3.25 [0.67], *p* < 0.001). Interestingly, participants with admin posts showed higher resilience (Md [IQR] = 3.17 [0.83]) compared to those who were not holding any admin post (Md [IQR] = 3.00 [0.50], *p* < 0.001). In addition, those who were involved in administration duties for more than 6 h a week were also more resilient (Md [IQR] = 3.17 [0.83]) compared to those with no involvement in any administration duties (Md [IQR] = 3.00 [0.67], *p* < 0.013).

#### 3.4.3 Institution type and resources

In terms of institution type, those who were working in a public university were more exhausted (Md [IQR] = 21.00 [5.00], *p* < 0.001) than those who were working in a private college (Md [IQR] = 19.00 [5.00], *p* < 0.001) or private university (Md [IQR] = 19.00 [4.00], *p* < 0.001). Participants who believed that their institution had sufficient remote learning resources were less disengaged and less exhausted (OLBI-D: Md [IQR] = 18.00 [4.00]; OLBI-E: Md [IQR] = 20.00 [4.00]) compared to those who believed that remote learning resources were partially sufficient (OLBI-D: Md [IQR] = 19.00 [4.00], *p* < 0.001; OLBI-E: Md [IQR] = 21.00 [4.00], *p* < 0.001) or insufficient (OLBI-D: Md [IQR] = 19.00 [3.00], *p* = 0.001; OLBI-D: Md [IQR] = 20.00 [5.00], *p* = 0.027). More detailed results are presented in Table 4.

**Table 4:** Comparison of OLBI-D, OLBI-E, BRS, and BTS based on the sociodemographic and work-related factors of the participants (*N* = 505).

	Disengagement	Exhaustion	BRS	BTS
Age	0.119	**0.003** ^**a**^	**<0.001** ^**a**^	0.309
Gender	0.257	**0.014** ^**b**^	**0.016** ^**b**^	0.473
Marital status	0.780	0.356	0.334	0.954
Having kids	0.194	0.055	0.311	0.74
Annual salary	0.638	0.873	0.96	0.540
Employment status	0.417	**0.023** ^**d**^	0.081	0.076
Academic rank	0.143	**<0.001** ^**d**^	**<0.001** ^**d**^	0.807
Institution type	0.143	**<0.001** ^**d**^	0.638	0.486
Faculty	0.325	0.560	0.881	0.097
Length of service	0.500	0.539	0.138	0.154
Hours of clinical setting work	0.719	0.055	0.225	0.053
Hours spent on research in a week	0.679	0.055	0.292	0.598
Hours of postgraduate supervision	0.354	0.133	0.837	0.054
Administration post	0.374	0.411	**0.001** ^**c**^	0.816
Hours involved in admin post	0.065	0.778	**0.024** ^**d**^	0.320
Familiarity with remote learning	0.392	0.404	0.208	0.852
Remote learning availability	**<0.001** ^**d**^	**<0.001** ^**d**^	**<0.001** ^**d**^	0.093

Significant *p* values are in bold. ^a^Pearson correlation, ^b^Independent-samples *t*-test, ^c^Mann Whitney U-test, and ^d^Kruskal Wallis test.

### 3.5 Correlations between burnout, resilience, and thriving

**Figure 3:**
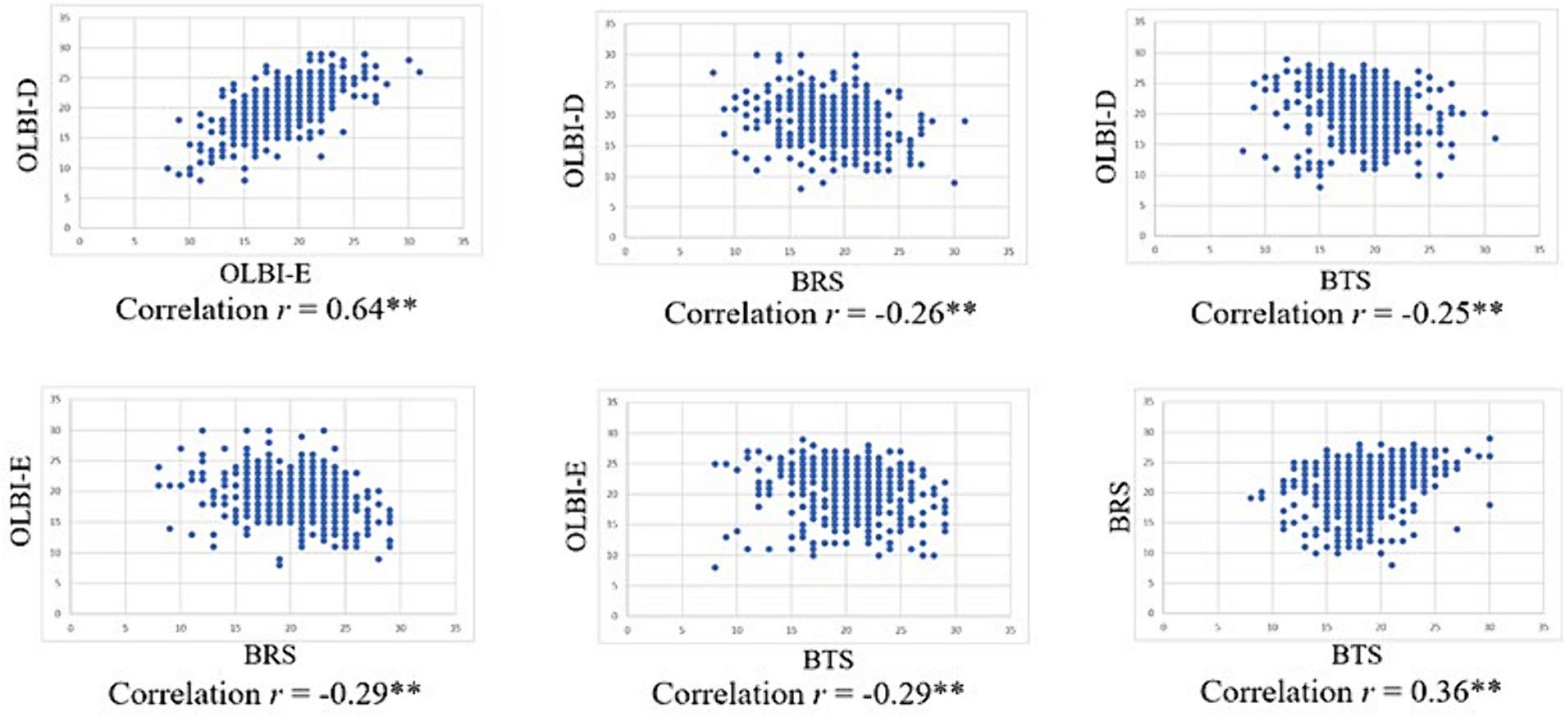
Correlation (Spearman’s Rank-order) for burnout, resilience, and thriving scores.

Spearman’s rank-order correlation analysis revealed a significant positive correlation between the OLBI-D and OLBI-E scores and a significant negative correlation between OLBI-D and both the BRS and BTS scores. OLBI-E scores were also significantly and negatively correlated with both BRS and BTS. BRS and BTS scores were significantly and positively correlated (Figure 3).

## 4 Discussion

The present study assessed the prevalence of burnout, resilience, and thriving among a relatively large sample of health sciences academicians from over 10 countries. Burnout, resilience, and thriving are all important concepts for academic health professionals and can impact the quality of health education delivered to future generations of healthcare practitioners. Burnout is a state of physical, emotional, and mental exhaustion caused by excessive and prolonged stress, a relatively increasing trend in academia (8). The present study’s findings showed a prevalence of high burnout at approximately 11%, affected predominantly by exhaustion over disengagement domains. A study conducted in Brazil among university professors recruited a smaller sample and used a different burnout assessment tool, underpinning a relatively higher prevalence of 40% (3). Another systematic review summarized burnout data from 12 studies conducted between 2005 and 2020. It reported an overall burnout risk of 37%, which is always higher than ours, but different assessment tools have been used (4). Considering that high burnout in our study means an increased risk of exhaustion and disengagement, this could expose academic staff to a state of losing interest, passion, and the ability to guide the young generation of healthcare practitioners adequately.

There have been several factors contributing to burnout in workplaces such as universities; these could include mainly job demands and lack of job resources, whereas available job resources exclusively predict engagement (3); burnout is related to health problems as well as to turnover intention, whereas engagement is related only to the latter (4); burnout mediates the relationship between job demands and health problems, whereas engagement mediates the relationship between job resources and turnover intention.

Our study participants had a medium level of resilience and thriving attributes. Resilience is essential in preventing burnout, as resilient people can cope with stress and adversity, bounce back from setbacks, and maintain a positive outlook (22). Our study revealed an inverse correlation between resilience and burnout. Similarly, a recent meta-analysis involving 29 studies conducted among nurses highlighted the inverse correlation between resilience burnout and exhaustion (23). This could pave the way for interventions to boost resilience and decrease the burnout risk among healthcare providers in academia.

Concerning the correlation between age and gender with burnout and resilience levels, our study showed that the younger generation and the female gender were more likely to be exhausted. At the same time, relatively older males were more likely to have higher resilience levels. Similar to our findings, an Italian study among healthcare workers highlighted that burnout was predicted by lower age, female gender, and low resilience levels (24). In this context, a relatively large United States study conducted among physicians underpinned that the link between female gender and burnout can be less significant by assuring equal treatment regarding work environment diversity and inclusion and perceived appreciation (25). These could be considered modifiable factors to reduce the risk of burnout among female workers. On the other hand, a previous Turkish study assessed resilience levels among teachers but did not show a significant association with either gender or age (26).

Furthermore, a study among university teachers in the Philippines indicated a moderate level of burnout independent of age, gender, academic rank, and service length (27). However, our findings showed a difference in burnout and resilience based on employment status and academic rank, where contact-based academics tended to be less exhausted than their permanent counterparts. At the beginning of their academic careers, instructors tend to be less resilient than their colleagues with higher academic ranks. This could imply the need for proper mentorship and advising programs for young academicians with proper support to set realistic expectations and goals while starting their academic careers. In addition, the present study showed that respondents from public universities tended to be more exhausted than those from private institutions. A previous large study among students highlighted that public university students tend to have lower odds of mental well-being (11). It is believed that the institution type could reflect the strategies followed to ensure staff well-being and allow them to adapt and grow professionally as successful, resilient teachers. In this domain, public universities in the participating countries might consider revisiting their staff support and well-being agendas with a periodical assessment of burnout and resilience levels.

Measures to alleviate burnout among academics should consider several factors contributing to burnout, such as job demands and lack of job resources (28). These could entail a reduced workload, which might be accomplished by increasing faculty numbers, decreasing class sizes, or providing more equitable work-life arrangements (29). This point is critical to consider, given that ethical climate dimensions such as role overload and clarity directly affect burnout risk, particularly for relatively younger employees (30). Further evidence supported a relationship between ethical leadership focusing on role clarity and burnout, encouraging adopting more active leadership to mitigate burnout risk (31). In addition, it is important to develop and maintain a supportive environment that provides opportunities for professional development, mentoring, and peer support (32). Finally, clear policies should be implemented to achieve work-life balance, such as setting boundaries, taking breaks, and regular exercise (29). In addition, various interventions could be considered to boost resilience, such as encouraging individuals to have a positive outlook on overcoming obstacles and accomplishing their goals (33). In addition, they can establish reliable social support networks upon which they can rely in times of difficulty (29). Lastly, a recent meta-analysis showed that resilience could be developed through interventions based on cognitive behavioral therapy, mindfulness interventions, and a mix of both (34). Moreover, to achieve a thriving, which is a state of being successful and thriving, academics in the health sciences should find a work-life balance, set realistic goals, work toward them with the necessary determination for academic success (35), and give back to their community by volunteering their time and energy to help the community improve. The latter should help acquire a sense of self-actualisation and a belief that the benefits of academic expertise extend beyond typical academic pursuits by integrating these benefits into students’ activities (36).

Finally, burnout, resilience, and thriving are all important concepts for health sciences academicians. By understanding these concepts and taking steps to prevent burnout, increase resilience, and thrive, health sciences academics can improve their physical and mental health, increase productivity, and achieve their goals. This could directly impact the quality and outcomes of higher education in the health sciences. We believe that the current study was able to present responses gathered from different international perspectives with a relatively large sample. In addition, it provided updated insights on the status of burnout, resilience, and thriving among a unique group of academic educators involved in health sciences programs. The findings of the present study could be used as a foundation for designing interventions and initiatives to support health sciences academics thriving and achieving their professional goals through resilience at minimal risk of burnout.

The present study has several limitations. First, there was a disproportionate distribution of responses among different countries, which did not enable cross-country comparison or country-specific recommendations. Second, the cross-sectional study has time-restricted limitations that do not allow for further investigation of potential causal relationships. Lastly, the online survey did not account for investigating the presence of any specific measures by academic institutions to assess or improve the well-being of their academic staff.

## 5 Conclusion

This study presents findings on the varying degrees of burnout, resilience, and thriving experienced by academic health professionals which are influenced by various sociodemographic and work-related factors. Interventions aimed at enhancing resilience and facilitating thriving may diminish the risk of burnout and enhance engagement among academic staff in the health professions field. Ultimately, this could directly impact the quality and outcomes of higher education in this field.

## Data availability statement

The raw data supporting the conclusions of this article will be made available by the authors, without undue reservation.

## Ethics statement

The studies involving humans were approved by the Research and Ethics Committee at the International Islamic University Malaysia (IIUM) approved the study protocol (IREC 2022-391). The study was conducted in accordance with the local legislation and institutional requirements. The participants provided their written informed consent to participate in this study.

## Author contributions

AN: Conceptualization, Data curation, Formal analysis, Methodology, Validation, Writing – original draft, Writing – review & editing. MoE: Conceptualization, Funding acquisition, Investigation, Methodology, Project administration, Resources, Supervision, Validation, Writing – original draft, Writing – review & editing. NM: Investigation, Project administration, Writing – review & editing. MK: Investigation, Project administration, Validation, Writing – review & editing. MB: Investigation, Project administration, Validation, Writing – review & editing. EF: Investigation, Project administration, Writing – review & editing. LK: Investigation, Project administration, Validation, Writing – review & editing. DR: Data curation, Investigation, Project administration, Validation, Writing – review & editing. AJ: Investigation, Project administration, Writing – review & editing. MAk: Investigation, Writing – review & editing. AbA: Investigation, Writing – review & editing. SC: Investigation, Project administration, Writing – review & editing. IM: Investigation, Project administration, Writing – review & editing. IJ: Project administration, Resources, Writing – review & editing. AhA: Investigation, Project administration, Writing – review & editing. AC: Investigation, Project administration, Writing – review & editing. MaE: Investigation, Project administration, Writing – review & editing.
